# “Paradoxes of Interdependence and Dependence”: A qualitative study of economic difficulties and relational encounters prior to men’s suicide in Ghana

**DOI:** 10.1080/17482631.2023.2225935

**Published:** 2023-06-22

**Authors:** Johnny Andoh-Arthur

**Affiliations:** a Department of Psychology, University of Ghana, Legon, Accra, Ghana; b Center for Suicide and Violence Research (CSVR), University of Ghana, Legon, Accra, Ghana; c Community and Life Empowerment Advocacy Network, Accra, Ghana (CLEAN-GH)

**Keywords:** Suicide, Men, Economic challenges, Dependence, Ghana

## Abstract

**Purpose:**

This study aims to explore the relational encounters that are shaped by economic difficulties prior to the suicides of men in Ghana.

**Method:**

Using a qualitative study design, and with the aid of a semi-structured interview guide, data were collected from 21 close relatives of nine men who took their lives in Ghana.

**Results/Findings:**

A Reflective Thematic analysis (RTA) showed themes reflecting four relational tensions corresponding to unique demographic profiles and circumstances of economic dependence on others: from dependence to independence; from control to living with and on others; from provider to dependence; and regaining control in a dependent relational context.

**Conclusion:**

The men’s economic challenges produce paradoxes of interdependence and dependence in that the interdependent social ethic enjoins persons in crises to disclose or seek help from close relations, yet for some men, doing so often draws social taunts, which further taint the social image of these men and contribute to suicides. Increased public education is needed to change unhealthy gender norms that affect men in social and economic adversity. Provision of practical economic support for men in economic and financial adversities is highly recommended.

## Introduction

Suicide is a complex behaviour that is widely regarded as a significant public health issue across the globe (Global Burden of Disease Self-Harm Collaborators, Naghavi, 2019). It occurs at the juncture of multiple factors: psychiatric, psychological, biological, social, cultural, economic, and existential, among others (Franklin et al., 2017; O’Connor and Nock, 2014; Platt, 2017; Shneidman, 1985). Despite its multifaceted nature, psychiatric factors have received much attention as far as understanding, treating and preventing suicidal behaviours are concerned. Recent years have seen an upward trend of scholarly interest in the roles non-psychiatric factors play in suicides towards a deeper understanding of effective remediation. For example, suicide is viewed by some as a deeply gendered phenomenon (Chandler, 2021, Meissner, Bantjes, and Kagee, 2016, Ramesh et al., 2022, Richardson, Robb, O’Connor, 2021). This view now prompts the treatment of gender as an explanatory variable in suicide studies away from the tendency in previous studies to treat gender merely as a descriptive factor (Payne, Swami, and Stanistreet, 2008, Platt 2017)

## Gender and suicidal behaviour

Across most countries, male suicides are up to between 3 and 7.5 times that of females even though suicide ideation and attempt are highly frequent for females (Bilsker, Fogarty, and Wakefield, 2018, Global Burden of Disease Self-Harm Collaborators, Naghavi, 2019, WHO, 2014). The term *gender paradox* has been coined to describe the differential prevalence of suicide mortality and morbidity for males and females, respectively (Canetto and Sakinofsky, 1998). Accounting for the phenomenon of gender paradox in suicidal behaviours includes biological differences between males and females, differences in early life experiences, social/cultural norms guiding emotional experience and emotional expression, coping, help seeking, and differences in suicide method selection between the two genders (Chandler, 2019, Moscicki, 1994). On the biology, it is suggested that testosterone, which is linked to impulsivity and aggression, is about ten times more in males than in females. The high propensity for males to engage in risky behaviours including aggression towards others and to themselves is therefore attributed to high testosterone levels in males (Sher, 2015). Within the non-biological explanations is a perspective that connects high male suicide mortality to gender stereotypes and role socialization. The prevailing view, according to this perspective, is that males are socialized to live according to what society expects of “men”. Ironically, the very societal expectation that seemingly connect to mens’ valued social identities also presents risk for suicide in some men who may find it difficult to meet such expectations (Kong, 2021). For example, studies have highlighted “stoicism” as a masculine norm that acts as a barrier to help seeking in traditional masculine environments (Broadbent and Papadopoulos, 2014). Furthermore, stoicism and self-reliance have been found to contribute to Australian men’s experiences of social isolation, which in turn, connected to their health problems including suicidal behaviours (Player et al., 2015). Similarly, dominant masculinity discourses, according to past and recent studies, reveal how help-seeking, for some men, is only contemplated following pain, endurance, stoicism and visible injury’ (Johnson, et al., 2012, O’Brien et al. 2005). In her study of 52 Irish men, Anne Cleary (2012) implicated the prevailing masculinity of the context in the men’s inability to identify symptoms and making disclosures despite emotional pains they experienced. Barriers to help seeking in men are found in other studies to be linked to fear of stigma, intense shame and perceived feelings of weakness (Rasmussen, 2017, Richardson et al, 2021). Thus, the degree to which males subscribe to such socially prescribed rule about what it means to be a man influences behavioural outcomes such as suicides. A longitudinal study has for instance reported a likely 34% greater odds of suicidal ideation among men who strongly identified with a key Western masculine role norm of being “self-reliant” (Pirkis et al., 2017). Strongly held gender norms manifesting as restricted emotionality, conflict between relations have been found in previous studies to pose risks for depression and low help seeking (Good, Dell, and Mintz, 1989, Leong and Zachar, 1999); two key risk factors for suicides.

## Theoretical considerations

Suicide is a multidimensional phenomenon that is not reducible to a single explanation (Shneidman, 1985). For men’s suicide, in particular, theoretical positions have largely coalesced around the concepts of gender, power, sex role, and masculinities. Critical analyses of masculinity have implicated the sociocultural context in the disturbingly higher rates of suicide among men in many societies (Khan, Ratele, & Arendse, 2020). This article focusing on the relational dynamics in the aftermath of job losses, and how they shaped the suicide of men is grounded mainly in Connell’s gender theory. According to Connell (1995) “[m]asculinity and femininity are inherently relational concepts, which have meaning in relation to each other, as a social demarcation and a cultural opposition” (p. 44). Accordingly, the author postulated three structures that characterize the gendered relationships between men and women, namely *the sexual division of labour* (gendered allocation of men and women to work and occupational roles), *the sexual division of power* (inequalities between men and women in the exercise of power and authority), and *the structure of cathexis* (the structure of affective attachments and social norms). In the opinion of Connell, the three structures exist at the societal and institutional levels with these social structures firmly embedded at the societal level through numerous historical and sociopolitical forces. Later work of Connell saw the addition of a fourth structure called *symbolization*, which depicted men’s control of social institutions and higher status given to their roles within social institutions (Connell, 2002). Connell also argued for the existence of collectively held understanding of ideal male practices in social settings. The author used the concept *hegemonic masculinity*, to describe an *idealized* form of masculinity at a given place and time (Connell, 1995). Notwithstanding, there is the acknowledgement of more than one kind of masculinity existing concurrently in societies (Connell and Messerschmidt, 2005). What is considered “masculine” may be different according to race, class, ethnicity, sexuality, and even gender. When desirable, some men adopt an idealized form of masculinity that is salient in a specific context; when undesirable, same men can distance themselves strategically from a previously adopted masculinity form. The talk of masculinity, according to Connell and Messerschmidt (2005), “represents not a certain type of man but, rather, a way that men position themselves through discursive practices” (p.841). Hegemonic masculinity has been investigated largely within the area of men’s health and men’s suicide in particular and scholars have implicated male role socialization process in risk behaviours among men. For instance, conformity to some masculine norms is found to lead to problem behaviours including substance use and suicides (Chandler, 2021, Lash, Copenhaver, & Eisler, 1998).

Joseph Pleck’s gender role strain paradigm helps to accentuate the nexus between gender norms and problem behaviours including suicides. To Pleck (1995), the gender norms for men could yield three different role strains: men’s failure to live up to internalized manhood ideal which among contemporary men is often a close approximation of the traditional code (*the discrepancy strain*); negative side effects men themselves and their close relations face when men fulfil the requirement of the male code due to desirable qualities they perceive in the male code (*the dysfunction strain)*. Pleck proposes the third, *trauma strain*, which to him, results from the ordeal of the male role socialization process. Krugman (1995), in his reflections on Pleck’s work, categorized male-role strain, with its grounding in feelings of inadequacy and inferiority, as a shame-based experience—“an experience emanating from a judgement against the self, which can provoke anxieties and lead to avoidant behaviours including isolation and suicide” (Andoh-Arthur, 2019). Recent evidence has shown that shame-proneness uniquely predicted concurrent social anxieties (Carpenter, Stebbins, Fraga, & Erickson, 2022), which some scholars have long drawn its connection to suicide (Hastings, Northman, & Tangney, 2000). In his Interpersonal Psychology Theory of Suicide, Thomas Joiner (1999) proposed two main constructs: *desire to die* and *ability to die* as underlying suicides. On *desire to die*, the theory connects it to two psychological states: thwarted belongingness/social alienation and perceived burdensomeness. He implicates acquired capability to die as a motive underlying the *ability to die*. The theory hypothesizes that when individuals have a weakened sense of social belonging and also perceived themselves to be a burden on others, *their desire to die* can be heightened. Suicide, according to this theory, is likely to happen in the context of *a desire to die* when people have acquired the capability to die through prior engagement in self-injurious and destructive behaviours such as suicide attempts, substance use, violence, etc.

## Patriarchy, employment, control and suicide in men

Patriarchy connotes authority: men in control of others. Patriarchy is a widespread social system across cultures. Men’s aspiration to be in control, coupled with societal expectation for men to be in control often puts some men in situations of extreme anxieties especially when there is a direct or indirect threat to being in control (Kong, 2021). For men who are married and have children, the immediate family context provides a vital arena for testing, exercising and maintaining control. A study by Gibbs, Sikweyiya, and Jewkes (2014) among young men living in urban informal settlements in South Africa revealed, for instance, that young men’s aspiration to traditional masculinity was closely tied to provision for a family and partner and control over them. Impliedly, potential threats to control might usually originate from and impacts closest dependents. Success at work and employment domains is fundamental for achieving control quests as it enables men to fulfil a critical masculine norm of providing for their dependents.

Especially for working-class men, meeting the needs of immediate family is central to “being a man” (Milner et al., 2017). Rivers’ (2014) study in Australia found that employment and work enable men to assert control in arenas of occupation and reproduction of patriarchal domestic structure such that tensions in these arenas inscribed the suicidal behaviours of some men (River, 2014).

## Communitarian system, patriarchy, unemployment and suicides in Africa and Ghana

Societies in Africa, especially those south of the Sahara are intensely communitarian with an ethical system that is also interdependently driven. Personhood within such systems is conceived to have social intentionality and is attainable through one’s relations with others (Adjei, 2019, Gavi et al., 2022, Menkiti, 1984). This ethical system is thought to foster mutuality, reciprocal obligations, and help seeking since individuals are enjoined to relate with others and to disclose problems to others in order to receive help. In Ghana, mutuality and reciprocal obligation are captured in the Akan language through such axioms as *Hu me ni so ma me nti na atwe mienu nam*. (To wit: it is for the purpose of helping each other that antelopes move in pairs). Help seeking through interdependent living is also expressed in an Akan language as “*sε etƆn woyare a, ennya n’ano edur*” (To wit: if you disclose your sickness, you will get the right medication for it). While these are ingrained within the socializing system as key values in Africa and in Ghana in particular, patriarchy, which is deeply embedded in Africa, appears to shape how to become a man as well as delimit emotional expression and the operationalization of the values of disclosure of problems differently for men. The patriarchal nature of societies in Africa, makes being economically independent a key social expectation of being a man. Key requirements for attaining valued social identity as a man in most societies in Africa include attaining some level of financial independence, being employed, having a regular income, and subsequently starting a family (Barker and Ricardo, 2005). These gender norms are well ingrained within the socialization practices of Africans which starts right from the family. As a major institution, the family is essential for “carrying out essential production, consumption, reproduction, and accumulation functions that are associated with the social and economic empowerment of individuals and societies” (Mokomane, 2012, p. 2). Key pathways to these functions, according to the author, include family capital and family resilience. There exists traditional division of labour along gender lines within the socialization processes of children in African societies (Shefer et al., 2007). These processes engender gender specific roles for boys and girls as far as family capital and family resilience building processes are concerned. Usually, rites of passage are used to reinforce clear demarcation between children, or boys, and men, and between men and women. In most traditional societies of Africa, there is the expectation for boys to be separated from their mothers, from female confines and quarters, and even to refuse to take on tasks that are considered female-home chores like washing, fetching water etc. (Barker and Ricardo, 2005). The refusal to undertake some household chores by boys likely produces tensions between boys and their parents as far as boys’ quest for independence and their obligations to family duties are concerned. Boys were expected rather to engage in social apprenticeship with their fathers to understudy the rudiments of farming, hunting, fishing that are essential to family capital building (Nsamenang, 2006). Home bound activities are reserved for girls and as revealed by Adomako Ampofo and Boateng (2007) in their study of boys in Ghana, the concept of male role in housework as one of helping, as doing a favour or kindness to a woman, was firmly lodged in the ideology of many boys they studied. An extract from one of the boys in Adomako Ampofo and Boateng’s (2007) work was quite illustrative of the gender demarcation within Ghanaian socialization of children thus: “I am a boy so I do not carry rubbish to the dumpster or cook in the kitchen; my sister however, can carry rubbish to the dumpster and she can cook” (p.62). These gender demarcations are also seen in the experience, expression, and disclosure of emotional problems in males. Thus in Ghana, there exists local scripts that highlights male power (e.g., *Ɔbarima na enom eduro a εye nwono*; it’s a real man who drinks a bitter medicine), male invincibility (e.g., *etuo toa esi Ɔbarima bo*- it’s a real man who uses the chest to face the bullet), male superiority over female (*Ɔbaa tƆ etuo a, esi Ɔbarima danmu*-when a woman buys a firearm, she keeps it in a man’s room), male non-disclosure (e.g., Ɔ*barima nkasa bebiree*- a real man does not talk much), and emotional restriction in males (*Obarima nsu*- a real man does not cry). These and many others are examples of male-related scripts that appear to emphasize male norms and yet have grave implications on male wellbeing and health. Typically, a male child who runs home to complain of having been beaten by another male is likely to be punished or taunted for not being able to fight back against the aggressor.

The transition from being a single boy to a married man is a status enhancer for men. It provides among other things means for offsetting perceived feminine attributes and household duties during boyhood and thus becoming independent and being in control, taking on responsibilities for, and affirming oneself as a real man (Adjei, 2015). The concept of “bride price”, in some African societies including Ghana, for instance, requires men to pay some amount of money to would-be in-laws as a precondition for marrying a woman. Ability to pay bride price is symbolically tied to economic and/or financial independence as well as indexes a man’s readiness to meet the needs of a potential spouse and the children who may be born within the marriage. Lack of ability to pay bride price before marriage or to meet the needs of spouse and children while in marriage destabilizes most men while exposing them to crisis including suicides. Studies have illuminated the roles unemployment, loss of income, and financial difficulties play in men suicides in Africa. A qualitative study in Uganda found loss of ability to work as a critical factor that heightened distresses and preceded the suicides among men displaced in wars (Kizza, Knizek, Kinyanda, & Hjelmeland, 2012). “The WHO (2020) reports that approximately 1993 suicides occur in Ghana annually. A 4-year suicide attempt trends in Ghana have also revealed that 707 suicide attempts occurred in 2018, 880 attempts in 2019, and 777 attempts in 2020 with 417 attempts recorded as of June 2021 (Akotia, 2023). Studies continue to reveal a disproportionate higher number of males in both suicide and attempted suicides in Ghana which makes suicidal behaviour in Ghana a predominantly male problem (Abdulai, 2020, Adinkrah, 2012, Quarshie, Osafo, Akotia, & Peprah, 2015) Studies in Ghana have revealed how job losses in particular exact heavy tolls on men’s wellbeing and contributes to suicides (Adinkrah, 2012, Kong, 2021).

Thus far, a rich corpus of evidence has been built to emphasize the problem of male suicide in and out of Ghana and the critical role job losses, unemployment and lost financial control play in men’s suicide both within and outside Africa. However, much is not known in terms of how the loss of job and income by men (some of whom are mostly breadwinners/providers) shape the relational contexts of these men and their close relations, and subsequently create a dynamic shaping their suicides. One of the closest studies to this line of research is the work of Gibbs et al., (2014) that examined how black men in informal urban settlements in South Africa constructed respect and masculinity from their closest dependents in the context of poverty and unemployment. However, the study did not have a focus on suicides. This article, being a part of a main study on men’s suicide in Ghana, explores the experiences of nine men and their relational encounters following unemployment and job crisis prior to their suicide. It is anticipated that such knowledge will help guide intervention for men who lose employment as well as their dependents towards early risk identification and effective help for these men and their close relations.

## Materials and methods

This study was part of a qualitative study exploring cultural meanings of suicide among men in Ghana. Situated within a social constructionist perspective, the study adopted a critical perspective and viewed the account of close relations of the deceased men as key sites for uncovering the men’s experience and relational encounters following unemployment, job losses and crisis prior to their suicides.

### Sampling

For the purpose of the study, accounts of 21 close relations of nine men who took their lives between 2014 and 2015 were analysed. In keeping with the research question; *what are the relational experiences of men following financial difficulties, unemployment and job crises prior to their suicide?* data of nine men who took their lives were analysed because they were information-rich sample (Malterud, Siersma, & Guassora, 2016) that illuminated how financial difficulties, prolonged unemployment or experiences of job loss had influenced their lives, relational encounters with their close relations, and their suicides. These nine were aged between 19 and 47; five were single while four were married with children. See Table I for their sociodemographic information. In Table I and throughout the manuscript, pseudonyms are used instead of real names of the deceased persons to protect their identities.

**Table I.** Sociodemographic information of the deceased.

Name	Age	Ethnicity	Occupation	Occupational status	Marital Status	Number of Children	Educational Level	Suicide method	Lived with
Odai	19	Akan	High School graduate	Unemployed	Single	None	High School	Hanging	Mother and Sibling
Waja	22	Akan	High school graduate	Unemployed	Single	None	High School	Hanging	Senior brother and his family
Tei	27	Ga-Adangbe	Factory Assistant	Unemployed	Single	None	Junior High	Hanging	Maternal Aunties and cousins
Saba	29	Akan	High School graduate	Unemployed	Single	None	Junior High	Hanging	Paternal Grandmother
Nunoo	34	Ga-Adangbe	Administrative Officer	Unemployed	Single	None	Tertiary	Hanging	Mother and sibling
Danso	40	Ewe	Businessman	Unemployed	Married	5	High School	Hanging	Wife and children
Kwabena	44	Ewe	Businessman	Unemployed	Married	3	Junior High	Hanging	Wife and children
Gbortei	47	Ga-Adangbe	Financial Officer	Crisis with current job	Married	4	Tertiary	Selfimmolation	Wife and children
Kwasi	36	Ewe	Auto Mechanic	Crisis with current job	Married	None	Primary	Stabbing	Wife

The 21 participants were aged between 24 and 80 and comprised four spouses, two parents, 6 each for family relations and siblings, and 3 friends (See demographic information of participants in Table II.

**Table II.** Sociodemographic background of participants.

Relation	Males	Female	Total	Age Range	Religion	Occupation	Education
Spouses		4	4	24–50	All Christans	3 petty traders 1 civil servant	1 Tertiary 3 Elementary
Parents		2	2	50–80	All Christans	1 retired public servant 1 Petty Trader	1 Tertiary 1 Elementary
Family relations		5	6	29–46	All Christians	3 Traders1 Pensioner2 Busineman/woman	1 Tertiary 4 Elementary 1. No school
Siblings	3	3	6	28–59	All Christians	1 Physician 5 businessmen/women	1 Tertiary 5 Elementary
Friends	2	1	3	24–56	All Christians	2 Businessmen 1 Trader	1 High school 1 No School 1 Elementary
							
TOTAL			21				

The recruitment of the participants for the main study was facilitated by the Homicide Unit of the Ghana Police Service. The resort to the police, for such a sensitive study, was occasioned by reasons including absence of national registry for suicide in Ghana and stigma against suicidal behaviours. When a suicide occurs, the police investigate the death, and working closely with the coronal system, they certify suicidal deaths (Adinkrah, 2012). The unique role of the police in the investigation and certification of suicides, created a prior relationship with the bereaved family. The author therefore contacted the police, introduced the study to them and solicited their support in accessing data and also relatives and friends of the deceased. The social context of suicide in Ghana makes it extremely culturally insensitive as an outsider to access suicidal information directly from the families. Research on suicides have mostly relied on suicide reports that get to the attention of the police. The police are the main institution in Ghana for any recorded information on suicides, which makes them also the main repository for information connecting deceased persons to families. The author accessed the relatives and friends of the deceased through the police. However, aware of the sensitivity of the topic, potential ethical matters to be raised with the involvement of the police, a sensitization session was done with the personnel of the homicide unit. The session was to improve the knowledge and attitudes of the police as far as suicidal behaviour is concerned while the author also had the opportunity to know at first hand the challenges some personnel go through when investigating suicides. A key outcome of this collaborative effort was the design of a culturally sensitive protocol for accessing families to introduce the study to them. According to the protocol, the author could meet individual families after they had agreed to participate after being contacted first by the police. For those that were interested, the researcher met them and gave detailed explanation of the study and its purpose.

The police involvement posed some challenges such as initial fear and apprehension in some participants during the study. Nonetheless, it also provided therapeutic avenues, assured the bereaved of the credibility of both the research and the field researcher, as well as created a viable means of conducting such a study in the context. The process enhanced trust and confidence building and fostered disclosures on a topic that is tabooed and criminalized in the study context. Bereaved relations who volunteered to be interviewed provided oral consent after which times and venues that were convenient for them were agreed on and the interview scheduled. Given the sensitivity of the topic, the author made prior arrangement with a professional Clinical Psychologist to handle potential distresses that could likely emerge from the interviews. Even though this arrangement was made known to all the participants, all declined this service despite obvious difficulties some had when some painful memories were evoked during the interviews (Andoh-Arthur et al., 2018a, 2018b; Andoh-Arthur et al., 2020). The study received approval from the Ethics Board of the Author’s Home University (ECH: 069/14–15). All identifiable information linked to the deceased persons, their families and participants were removed in strict compliance to anonymity and confidentiality.

### Analysis

Seeking answers to the question *Why suicide* presents methodological conundrums for researchers because the very actors are deceased. For that reason, proxy accounts present alternative choices even though such accounts also do not accurately represent the actual motives of the deceased. Notwithstanding, scholars have argued for robust methods that rely on the accounts of different persons in different close relationships with the deceased such as spouse, children, parents, friends, other family relatives during the reconstruction of the suicides (Hjelmeland, et al., 2012). In line with Hjelmeland et al., (2012), this study gathered accounts from persons in close relationships with the deceased such as spouses, parents, siblings, friends, and some other family members of the deceased persons. Despite their inherent limitations, proxy accounts of this nature have offered some rich insights into suicides in other countries (Kiamanesh, Dieserud, and Haavind, 2015; Kizza, Hjelmeland, Kinyanda, and Knizek, 2012). Guided by the type of data, the Author used the Reflexive Thematic Analysis (RTA) (Braun and Clarke 2012) to analyse the accounts of the bereaved.

RTA was chosen because it is an easily accessible as well as a theoretically flexible interpretative approach that facilitates the identification and analysis of patterns or themes in a given data set (Braun and Clarke 2012). Accordingly, RTA is viewed as reflecting researcher’s interpretive analysis of data conducted at the intersection of the dataset, the theoretical assumptions of the analysis, and the analytical skills/resources of the researcher (Braun, Clarke, Hayfeld, and Terry, 2019, Byrne, 2021). Theoretical assumptions of RTA are conceptualized as a series of continua along essentialism and constructionism epistemologies; experiential and critical orientation to data; inductive versus deductive analyses, and; semantic versus latent coding of data (Byrne, 2021). The approach to analysis was grounded in constructionism, with a blended experiential and critical stance, even though priority was given to the latter. Giving priority to critical orientation was premised on the fact that data was attained through third persons who shared similar sociocultural contexts with the deceased men. These relatives also occupied different subject positions in the lives of the deceased and thus may be reflecting their own biases in their accounts. This way, accounts were seen through and interpreted within socio-historical context. The intention was to uncover and disrupt taken-for-granted issues shaping the construing of masculinity and at the same time limiting men’s psychological wellbeing. A purely inductive analysis at both semantic and latent levels was conducted. Subsequently, the author followed the six-phased analytic approach proposed by Braun-Clarke (Braun & Clarke, 2006, Braun et al., 2019): familiarization with the data, generation of initial codes, searching for themes, reviewing themes, defining and naming themes, and report production. At the familiarization stage, the author got intimately familiar with all the transcribed interviews by first engaging in an active listening of the original audio recordings. Next, he read and re-read all the transcribed data, at some points, verifying some of the written text by listening to the original audio recordings. The aim was also to achieve a better contextual understanding of the data. At the next stage, generating initial codes, the author, with the research question in mind, adopted a blend of latent and semantic coding to generate key initial codes that were very relevant to the aims of the research. Multiple codes that were generated in the previous stage of the analysis were critically examined by the author for their underlying meanings after which they were coalesced into about four themes. The naming and defining of the themes were facilitated mostly by a discernible, and an encapsulating story of transactional and conflictual social exchanges between the men and their closest relations. Depending on the men’s unique social and demographic situation, four different outcomes (themes) were produced. The analysis took place through iterative process of “dialogue” involving the author and the data.

### Findings

Data revealed that the social image and identity of the men remained defined through a give and take exchange loop. Within this loop, validation of status is seen through demonstrable responsiveness to others and the wider social context. While the transactional relationship is seen by some of the men as social insurance against an uncertain future, the loss of economic control and its attendant loss of ability to meet demands of others threatened social status, and eventually contributed to a state of dependency which many men found challenging to adjust to. The situation contributed to paradoxes of interdependence and dependence when the men’s survival hinged on dependence on close family relatives, yet doing so undermined their social, psychological and emotional resources, thereby creating the path for social withdrawal, avoidant behaviours and eventually suicide. These are elaborated below:

### From depending to independence: tensions between autonomy and obligations of depending

The experience concerns two young decedents who were depending on their family relations: older brother, and a mother respectively. The theme captures the tensions that characterized the decedents’ autonomous pursuits and the obligations circumscribed in the dependent relations. The decedents attempted some independence quests towards social recognition as boys and men. However, these independent quests appear to have collided with obligations of depending within their closest interpersonal domains. For example, a deceased, Waja (aged 22) who was a Senior High School graduate exploited his musical talents to survive thus: “He was someone who liked church. He even knew how to play drums very early so he had been visiting churches playing drums. Sometimes he survived on money from professional bands that hired him up for programmes” (older brother). Waja, thus actively worked to salvage his educational ambition. He decided to seek opportunities elsewhere and subsequently lost the financial support. He had some conflicts with his older brother on whom he depended over the decision to relocate to another place. This led to Waja’s open threat to harm as revealed by his brother. “He began threatening me that if I did not give him money to go back to where our parents lived, one day on my return from work, I would not hear good news”. True to his threat, the deceased left home angrily and subsequently killed himself nearby.

Another decedent, Odai (aged 19 and a graduate of Senior High School), had a stable dependent support from a single mother in meeting his educational needs. He extracted this support to his mother’s discomfort, and yet refused to honour familial chores that came with depending; thus,

He pestered me to pay 250 Ghana cedis (About 80 US Dollars then) as registration fee for a special class that could help him pass an exam for a scholarship award. I wasn’t having money then. I even told him to wait but he did not budge and kept pestering me”.

Education enhances life choices and affords power to people. The quote illustrates a determined young man in pursuit of his autonomous quests. Tensions started mounting between Odai and his mother when autonomous quest clashed with obligations in the dependent relations. To depend implies subjecting oneself to other’s control including being responsive, obedient and respectful. Contextually, failing to obey the one on whom a young person depends is perceived to have serious consequences as captured in a local axiom “*Abofra a onntsie no, ƆkƆ antseadze kurom*”. This literally translates as “a disobedient child eventually lands in the place of no return”. Odai’s perceived lack of responsiveness and apparent disobedience created incessant conflicts with his mother which preceded his suicide. The mother mentioned:

It was a Saturday evening; I came home to realize there was no water. I enquired why he didn’t fetch the water? In fact that day, I became furious with him. I said “I gave you money on Friday you didn’t fetch water. Saturday too you didn’t fetch water, why?” He kept assuring me he would fetch but he didn’t. We slept that night but when I woke up, I didn’t see my son and I exclaimed “Where is my son?” I rushed out and found a table at the balcony. I lifted my eye and saw my son hanging.

Gleaning from the above, these young men, by their circumstances, depended to meet critical needs. However, fulfilment of obligations circumscribed in the dependent relational context often collided with their autonomous pursuits, a situation that generated conflicts in the closest interpersonal dyads prior to the suicides. It is important to underscore that the deceased in the case above, according to the mother, took his life around the time he had suffered academic setback and also just few days after an elderly man in his neighbourhood had taken his life. Perhaps, it might be reasoned that events at the personal level and both within and outside the son-mother dyad had become what Community Psychologists will describe as *proximal personal and contextual risk* factors to an underlying vulnerability to suicide.

### From control to living with and on others: Threatening social image

The experience of three men who were already working but lost work is highlighted. These men aged between 27 to 34 years had not married nor had children, and were living with family relations. The theme captures distresses that accompanied loss of job while living with and on close family relations. Their experience was threatening to their social image.

Narratives from participants are replete with various ways the men achieved a sense of control prior to various adversities they suffered in the period just before they killed themselves. For three unmarried deceased men Tei, Saba, Nunoo (aged 27, 29 to 34 years, respectively) work afforded them choice in life and opportunity to pursue autonomous quests. The period following job loss and unemployment created tensions between them and close family relatives with whom and on whom they lived. A maternal Aunty said this about Tei:

He was very lazy. He didn’t go to work regularly. You will always see him in the room and when you ask him why he did not go to work; he would say there is no work to be done at the work place.

Tensions here arose from perceived availability of work and perceived non-availability of work on the parts of relatives and the deceased person, respectively. Herein is “victim blaming” as if to suggest that the deceased was intentionally refusing to work and that a man should find work to do no matter the situation. Upon Tei’s suicide, another maternal Auntie revealed:

I even got angry the day this incident (suicide) happened. People even said we have been starving him, etc, etc. For Christ sake, he was twenty-seven years. Must I keep on taking care of him?

At an employable age, and undergoing crisis with work, the second quote revealed a state of continued dependence on relations beyond socially acceptable age of dependence. Tei was being implicitly perceived as a burden.

A similar story is shared on the deceased person called Saba and aged 29 years. For his part, a stable familial support towards success in occupational domain was disrupted through unsuccessful autonomous quests. A paternal grandmother said

He always said he would like to be a doctor. Because of that ambition, the father did everything by educating him and placing him in an apprentice work when it was clear he (the deceased) was not interested in education. Every expense the boy made, the father paid; just to encourage him to realize his dream one day. He was the father’s only child. He was dismissed from the work. Hmmm. No one understands really what happened. It is all because of marijuana.

Consequently, Saba’s dependence became chronic, and his choices, increasingly maladaptive as the paternal grandmother further revealed

Anything he needed, he just asked me then I will ask him to go for money to buy what he needed. But I later realized whenever I gave him the money; he rather spent it on the drugs and not food. Sometimes too when I asked him to buy me food, he would take the money but would rather end up not buying the food for me but spend on drugs.

The maladaptive self-management style appeared to have also nourished a behaviour that was counter normative; dishonesty to one on whom the deceased depended. This is mostly likely to have sullied a prior healthy relationship.

Concerning Nunoo, aged 34, there was initial success with work occupation, which created a work life balance: “He woke up early, went to work, and came back and rested for the next day. He wasn’t troublesome at all” (Older sibling). Security attained through stable work helped create an enabling context for meeting own needs and also for maintaining healthy interrelationship with close family members “He wished to help always. My mother tried to complete this house we live in but she could not. My deceased brother helped. He built his own room in which he lived” (older brother). As long as Nunoo was able to extend support to family’s project, a healthy interdependent relation emerged. His older sibling further revealed “We were very close. We helped each other. I sometimes bought a gift for him and he also bought some for me”. However, job loss undermined personal balance, and contributed to dependency with its attendant distresses as found illustratively in the words of Nunoo’s biological mother.

Everything was fine until he lost his job. I was helping him to meet some of his needs. I knew it was hunger that makes people very disturbed, so I ensured that he was never hungry as I bought food for him. I had never anticipated that he would kill himself

Gleaning from the above, a healthy interdependent relation existed when he was working. However, Nunoo’s downward spiral began just when he lost his job and had to rely on others for survival. However, his close relations appeared to have been well disposed to responding to a cry for help as is expected of families within the interdependent social ethic. As illustrated in the quotes, Nunoo was provided for in a way as to ease the distresses from the job loss, but he went on to kill himself. The issue of economic control being a critical masculine norm in the patriarchal systems in Africa is well documented. Silberschmidt (2005), however, reveal the irony in this system is that male authority is grounded materially while male responsibilities are normatively constituted” (p. 195). Loss of economic control contributes to authority and image loss. Some studies in Ghana for instance reveal pejorative descriptors that are attributed to men who are not economically and financially dependent (Adjei, 2015). Such situation is found to contribute to suicidal behaviours especially for men who conform to such masculinity norms (Adinkrah, 2012). Nunoo may have identified with traditional masculine norms that are premised on men’s economic provision (so-called benevolent patriarchs) in relationships. That identification could make it difficult for him to understand the psychological impact of not accepting or seeking help during such distresses as also found in Cleary’s (2012) study. He might have interpreted continuous depending as embarrassing as was found also in Gibbs et al. (2014) study among South African young men living in urban informal settlements.

The theme, illustrate the tensions in the transition from being in control and falling back on others when job loss or job crisis sets in. Unlike in the previous theme, where tensions were more interpersonal, tensions in this theme were more intrapersonal as these men perceived a threatening social image with the dependent living they fell to.

### From provider to dependence: Threatened social image

The experience here concerns men who were married and had children. By their social position as husbands and fathers, a duty to provide for others crucially defined their social identity. However, the duty to provide clashed with the necessity to rather depend when they lost their means of livelihoods; job. This theme captures vividly the psychosocial tensions that characterized their living situation prior to their suicides.

Men who were married with children lived in a healthy interdependent relation with their close families and relations until job crisis surfaced and they had to depend. To illustrate the importance of work as precondition for assuming the role of a husband especially, the spouse of a 40 year old deceased person named Danso recounted the processes leading up to her marriage:

“Before he married me, I advised him that if he was doing his own business, my family would see that he was a responsible man. He started his own pub that was doing well, so he came to see my family. My family was pleased with him and gave us the go ahead to marry.

Given the precondition of work success before marriage, Danso, intent on marrying, had to succeed in business, presumably, to assure the would-be in-laws that he was capable of fulfiling a provider role as a man/husband. However, things did not go the way he expected when, in his bid to protect his business, he acquiesced to a family relative’s advice to seek spiritual support to boost the business which failed. His friend reveals;

“He had a successful business in the 1980s. Unfortunately, he followed his female cousin to seek spiritual protection to boost the business. It did not work and things started going bad. So it came to a point when he was saddled with huge debts.

The friend continued “His relatives visited him a lot when he was doing well in business but they stopped visiting when his problems started”. Business success afforded Danso much control and enhanced his symbolic capital beyond fulfilment of provider roles. Seeing that he lost both materially and socially due to a perceived misdirection from his female cousin, Danso, in protest, did not attend that cousin’s funeral when she died: “He was so angry with the cousin that he never stepped foot at the cousin’s funeral when she died” (spouse)

The importance of work success in indexing provider roles and the attendant distress emanating from job loss is seen in the case of Kwabena, a 44 year old deceased. His sister mentioned “My brother sold his car and gave part of the money to the wife as seed capital to start some business”. Selling a personal car, from the preceding quote, was Kwabena’s effort at fulfiling breadwinner (provider) roles. That is, he resourced his spouse to have a steady stream of income to meet daily needs at home. At some point, provider role extended into the arenas of affection to spouse and involved fathering, thus “he loved her (wife) so much and his children. He would bathe the baby; he will normally carry the baby”. The wife, a trader, corroborated “When I tell him I am not feeling fine, he would come and sell my wares and that would help him to do some exercise and play with the child”. Unfortunately, ill-health truncated Kwabena’s continued stay at work leading to a downward spiral. The wife further recounted that

“He fell ill and was asked to stop work. Some few days to his death, he became very withdrawn. He stopped cooking and caring for the children and was very reserved. The day before that incident (suicide), he was in the room the whole day. He never went out”.

Kwabena’s change of fortune affected him psychologically to the extent that he could not reconcile the present with the past as recounted by the wife “He usually said ‘Me of all people?’ then I will encourage him that he was having money previously but had no children but today he has a child so he should be happy”.

Gbotei, 47, was also helpful to others until he had job crisis. His spouse revealed that he was the type who found joy in helping people but was distressed when he could no longer help others due to job crisis “Things were not going so well with his job, it got to the extent that he could not help people the way he wanted to, and he was worried”.

Having economic power to be in control was prioritized over the very persons control was to be exercised on. As seen in the case of Kwabena, having the child at a relatively older age was not enough to compensate for the loss of work just as Gbotei was equally worried he could not help others any longer even though he had a supportive wife and children.

### Regaining control in dependent relational context: The paradox

The theme captures various ways the men who had fallen to dependence sought to gain control in that context. Their determined effort to reassert control saw them engage in masculine performances that rather undermined their resources and heightened both intra and interpersonal conflicts. This is mostly found in the situation of those with wives and children.

A prolonged stay in joblessness contributed to continued dependence on economic viable spouse mostly. However, these men engaged in practices which they might have perceived were essential for regaining some control. Danso’s spouse had this to say

“Any time I returned from work he would be stalking me to see where I will put my money so he can take it and use for drinking. He would not do any work and when you have worked and you give him food, he will not eat what you have served him. Even the money that I had kept for the upkeep of the kids, he would take it and go and mess himself with it. At night too, he would not let me sleep (excessive demands for sex). He will disturb me until daybreak and when morning breaks, he will sleep all day.

The quotes illustrate attempt by Danso to regain control through stalking spouse for money and substance use behaviours. Implied in the quote is also the use of sexual violence to reassert power over the wife. Spouses were main sources for displacing existential frustrations and reassertion of power. Dependence had come to mean survival regardless of its psychosocial impact on spouses and even children. Danso’s spouse further asserts “In a way, he had no problem because whether he got a job or not he would always come back to find food in the house”. Recounting her encounter with Danso moments prior to his death, she revealed in this illustrative quote,

Realizing his situation, I didn’t bother him with school fees. My children had exams and so I had paid their exams fees. I was on my way to a funeral when my husband called asking for money. I told him I was on my way to the funeral and I did not have money for him. He kept calling and I felt his call would disturb the other passengers in the car so I put both phones off. Soon as I put my phone on, I had a call from a neighborhood friend who told me my husband was found hanging in his room!

It is difficult to directly ascertain the actual reason for Danso’s suicide. However, the suicide, coming immediately on the heels of spousal inability to respond to a financial request, could be construed as a controlling behaviour. This is because the spouse recounted previous instances where the deceased threatened to take his life as a way to extort money for drinking, thus: “He has threatened to kill himself before when he needed money and I could not give him. Even if you give him money, the sad thing is that he will use it to mess himself in drinking” (spouse). It is documented that when some men are unable to achieve desired results through controlling behaviours, they seek a way to re-establish both the gender order and respectability through such acts as violence (Gibbs et al., 2014). In this case of Danso, the violence appears to have been turned inwards- towards the self.

Another man called Kwesi (aged 36) also fell to total dependence on his wife, but had extreme difficulty living in that condition. Not only did he openly voice his disquiet about relying on spousal support, he at times lied, or found unorthodox means of extracting cash in attempt to save face. The wife said

“I gave him money so that he would not be worrying too much. One day he wanted to go out and rebuild his collapsed workshop, I searched his pocket and there was no money so I asked him if he had money and he said yes. I asked him to show me the money he claimed he had. He only told me the money was somewhere“

The wife further shares

We saved our monies in different banks so sometimes, I gave him my money for safe keeping but because of these problems, he withdrew all the cash including mine which was with him for safe keeping. The very Friday when he died, he withdrew cash and sent to his mother. So according to those who found him at the time of the incident, he had 100GHC in his pocket that was after he had sent either 100 or 200 GHC to the mother. He was the only child of his mum. The bank receipt for that transaction was inside his pocket when he stabbed himself”.

Though he underwent extreme financial difficulties and depended solely on a spouse, his strategy for regaining control included being manipulative of the wife and taking unilateral decisions in spending family finances and even finances belonging solely to the spouse. Prior to his death, and being the only son of his mother, he felt a strong need to remit his mother as a way of reasserting control and preserving image of a provider in that context, and in line with the reciprocal obligations children are enjoined to have for their aged parents.

For Kwabena, when he realized that wifely dependence was no longer sustainable, due to the precarious economic situation of his wife at the time, he compelled the wife to engage in romantic solicitation on social media in order to get money to meet his medical bills. She admitted

“We did all these just to get some money for his upkeep. One day, a social media friend I was soliciting help from proposed the idea of me joining him in his country. My husband threatened me that I should not leave him and go anywhere.

In the case of Gbotei, 47, his wife alluded to his open remonstration over his continued dependence and the apparent role reversal that had taken place “He felt that due to the problems he was going through, there were some responsibilities I was not supposed to do as a wife that I was doing and all of those things got him worried” (spouse).

The theme reveals extreme inner struggle in these men who perceived limited options as far as existential survival was concerned within the context of marriage. They had to live with and on their economically viable spouses, however, the pressure to breakout of the cycle of dependence appeared to have pushed them to resort to masculine practices, which increasingly compounded their inner struggles and also heightened interpersonal conflicts with those spouses.

## Discussion

This study aimed at examining how loss of economic control inscribed the interpersonal relations between men and close relations prior to the men’s suicides. Findings reveal a transactional social system in which the men’s social identity crisis was occasioned by changing fortunes in the sphere of economic control. The situation paradoxically contributed to a situation where total dependence for survival contributed to emotional difficulties. The men’s attempt to salvage the situation saw them engage in masculine performances which, in turn, diminished their personal and social resources.

Putting this contextually, the social ethic of societies in Ghana enjoins family members, and most especially, biological parents to nurture the growth of children through provision of material and emotional support. The aim, among other things, is to raise responsible adults, who can competently live on their own, as well as being sources of mutual support for both older and the younger members of the society. Care provision is also seen as social insurance in that when children are raised and become successful, they will in turn, be obligated to also care for their benefactors (Andoh-Arthur, 2011). This value, bordering on reciprocal obligations, is enshrined in the popular adage that “*wona ahwε wo ma wose efifir a, wo so hwε no ma ne se nfifir*.” (if your mother cares for you to grow teeth, you are also to care for her till she loses all her teeth). Within the communitarian context, and especially within the matrilineal societies of Ghana, mother, in the above proverb is a metaphor for adult carers since it is believed that a child is everyone’s child (Nukunya, 2003). Thus, dependence is a default relational system for every young person growing up. However, as children grow up, gender role socialization during adolescence guide boys and girls towards feminine and masculine practices. Key among these practices is an orientation for boys towards shelving dependence to an independence ethic (Gyekye, 1995; Nukunya, 2003). As alluded to earlier, a young boy who returns home to complain of having being beaten by peers receives opprobrium and is requested to be a boy by fighting back, an indication that he should be in control of his own affairs.

In this study, young men (*n* = 2) who were depending on close family members encountered problems relating to the collision of autonomous quests and meeting of obligations inscribed within the dependent relational context such as obeying instructions from carers and meeting family chores. Gender demarcation in household chores reinforces an idea in some boys that certain chores are emasculating, and that boys who go out of their way to execute them are merely helping rather than see them as duties consistent with observations made by Adomako Ampofo and Boateng’s (2007) study. From early adulthood to middle adulthood, being in control of one’s life is highly valorized and is also a precondition for exerting power and influence in the immediate family community context (Nukunya, 2003). The status of independence not only grants affordances enhancing a man’s social and symbolic capital, it also obligates a duty to interconnect with others in a mutually beneficial ways. In that transactional loop, social embeddedness through provision of support to others and being dependent on is exchanged for social recognition.

Work occupation, marriage and family making provide safe arenas for enacting these practices towards becoming a real man. Job losses of three men, contributing to continued dependence on family relations in some cases, heightened conflict with close relations due to perceived burdensomeness while in others, the men socially withdrew prior to suicides.

For the men who were married and had children, marriage and family life context served as direct arenas for achieving ultimate status of masculinity, i.e., patriarchy: provision, procreation, and power to control others. For these men, accounts of success in their relational context were framed in the past when they recorded successes in occupational domains. That is, these men not only were in control of their own lives but also used the economic power derived in occupational successes to support close family members. They were therefore benevolent patriarchs in the past and sought to maintain this social image even in the present. However, job losses threatened control and weakened support networks for some.

It is worth noting that the job loss presented two possible outcomes to these men: to either bounce back to economic control (regain control) or regress to a state of total dependence on economic viable relations (lost control). Unfortunately, the former was not the case for any of the men whereas the later was the case for all the men; i.e., total dependence on economically viable relations. This created a paradox because while depending on economically viable spouses had a key existential value, it appeared to have also created existential angst in the men. The latter pushed the men to attempt regaining control through substance use, conflicts with spouses and other close relations, and suicides, some of which appeared to have been a direct action against spousal refusal to acquiesce to their demands, attempt to reassert power or escape from existential frustrations.

These findings of the association between financial difficulties, unemployment and job crisis and male suicides are consistent with previous studies. At the ecological level, increases in male suicidal behaviour have been linked to periods of economic uncertainty (Richardson et al, 2021; Vandoros et al., 2019). Further, a systematic review also found links between job loss, debt and financial difficulties and elevated risk of mental illness, self-harm and suicide (Moore et al, 2017). A number of qualitative studies have also thrown light on this observed link including Stack and Wasserman’s (2007) study which found proximal risks to male suicides to include loss of income and job-related problems. The importance of work problems to suicides is also highlighted in the sociological autopsy into men’s suicide in the United Kingdom (Scourfield, 2012, Shiner et al., 2009). For societies in Africa where strong patriarchal systems exist in the midst of weak to no welfare policies, the loss of job with attendant loss of income poses much greater risks for men (Adinkrah, 2012).

Economic and material provision for one’s family is a crucial obligation for men and constitute successful male identity in Ghana. Contrastingly, where women function as economic providers (as in the situation of role reversals)—such men are described as “useless man” (כbarima hunu) and may be subject to social stigma (Adinkrah, 2012, Adomako-Ampofo, Boateng, 2007). Failure to carry out socially expected obligations and roles are viewed as instances of personal irresponsibility, callousness, or generally vicious character and often lead to feelings of shame and eventually suicides (Adinkrah, 2012, Kong, 2021).

Male suicide in Ghana, according to Camilla Kong (2021) highlights a complex, potentially contradictory web of communitarian values that are thought to be central to African personhood, that is, what it means to be identified and recognized as a person with a certain moral status. Not fulfiling the criteria of personhood is morally blameworthy because it is antithetical to normative orientation towards oneself and one’s community (Kong 2021). It appeared that the men in this study valorized the *benevolent patriarch* identity and therefore felt strongly impelled to live it; consistent with internalized masculine norms to provide. However, the loss of job and job crisis occurring prior to their suicides constrained them from doing so. The situation might have presented *discrepancy strain* to them in line with Pleck (1995). Distresses faced prior to their suicides seemed to have been occasioned by perceived loss of respect, social abandonment, and social anxieties that were all connected to problems with one of the crucial social expectation that defined their identity as men: to provide. Even though ready support was made available for some of the men by their families, there were also instances where some of the men felt abandoned during their crisis. Within such a transactional social systems, the implicit rule for some (such as the families that abandoned their men) is that men are as important as their ability to provide for others despite their life circumstances. Such thinking goes against the grain of the humanistic ethic to support persons in need within communitarian societies (Gyekye, 1995). Consistent with Kong (2021), male suicide in Ghana reveals the incoherence within the communitarian conception of personhood. That is, the motivation for male suicides is not men’s repudiation of social responsibility or disregard for the community. Instead, “it is an intense sense of personal responsibility towards meeting prescribed social norms and roles associated with gender” (p.89). Seeing the above from the interpersonal theory of suicides (Joiner, 2005), the men in the study might have perceived being a burden on others given their continuous dependence. Increased sense of alienation and isolation due to their circumstances, and presumably, their acquired capability to harm themselves, through previous suicide attempts, substances use behaviours, etc., could have heightened both the desire and ability to die, in line with Joiner (2005). An intense psychological ache might have been unbearable for them for which reason they might have resorted to suicide as a way to escape (Shneidman, 1985).

It is important thus to view suicides generally, and male suicides more specifically as a crisis requiring help rather than a crime requiring punishments. However, within male crisis discourses, conservative narratives position high male suicide rates as a pernicious outcome of “threats” to traditional gender roles and norms, with its prescriptive return to these traditional gender norms as solution to male crisis. Contrastingly, progressive crisis accounts use male suicide to demonstrate that existing gender norms harm some men as well as women and argue for alteration of these norms to address male suicide (Jordan and Chandler, 2019). Following the progressive accounts, Kong (2021) has suggested *Critical Sankofa* as an analytical resource for understanding and addressing disruptive masculine norms that paradoxically underlies high male suicides in Ghana. As an Akan Adinkrah symbol: *Sankofa* is often depicted as a mythical bird flying forward while looking back, and is denotative of the idea that “going back to what is forgotten is not morally wrong”. In other words, sankofa communicates the “philosophy of retrieving lost or forgotten gems from the past as one moves forward; it involves reclaiming parts of African practice, history, and standpoints that have been hidden or distorted” (Kong, p.89). Critical Sankofa, according to Kong, is “a process of reclaiming cultural values through the critical evaluation which leads to the reflective endorsement or rejection of past practices or traditions” (p.90). In other words, critical sankofa involves the reflective sifting through, refining and pruning of, different aspects of the cultural past, to determine whether it warrants a place in the current scheme of things” (p.90). Guided by Kong’s thesis, this study calls for social changes in the way societies in Ghana and similar contexts construe men, masculinity and personhood in general. Worth noting within the progressive crisis discourse is the need to situate suicide generally and men’s suicide more particularly within intersectional frameworks. This is because some men and not all men engage in suicidal behaviour in keeping with Connell and Messerschmidt (2005) that more than one kind of masculinity exist concurrently in societies (Connell and Messerschmidt, 2005). The implication is that depending on the context of their peculiar experiences, individual men can be variously defined by the fact of their socioeconomic, class, sexuality, race, and cultural circumstances in ways that may increase or decrease their vulnerabilities to suicide and not just because they are men.

Men should therefore not be treated as a homogenous group and individual men not defined by changing economic circumstances of their lives. Rather, they should be defined by the intrinsic worth of being humans; beings who are not impervious to life adversities but can be assisted to overcome adversities. Societies and families must be there for men in economic adversities just as men are also expected to contribute to the family and societal good in good times and should see nothing wrong about depending on close relations in times of economic adversity. This symbiotic relationship ought to remain inviolable at all times. Perhaps, the finding of this study should inspire the need for critically interrogating the phenomenon of gender paradox of suicidal behaviours within cultures. It is documented that prior suicidal attempt is a single most important risk factor to suicides (WHO, 2014). For many countries, females dominate in depression and suicide attempts: key risk factors for suicides. One of the explanations for the higher attempts and lower suicides rates in females is the tendency for females to seek professional help for depression, especially mild to moderate depression (Moscicki, 1994, Shi, Yang, Zhao, et al., 2021). The higher suicide rates in men, particularly in times of economic adversities as found in this study, may reflect underlying distresses men face in such situations, distresses the symptoms of which might not directly map onto existing diagnostic criteria for depression. Societal expectation for men to restrict emotions embedded in cultural scripts such as “obarima nsu” (a man does not cry) might lead some men to find maladaptive ways to express and cope with their underlying distresses as Felicia Garcia (2016) also found in her ethnographic study of Irish men.

This study makes a strong recommendation for clinicians to be innovative and flexible in creating empathetic relationships with male clients towards getting them to disclose and cope with underlying problems. Further, the study strongly argues for a broadening of masculinity conceptions beyond narrow conceptions of material provision, sexual performance, procreation, and protection to include norms that give men several possibilities of being men irrespective of social and life circumstances, including being involved parents, seeking help and depending on others when in crisis, disclosing emotional vulnerabilities among others. While all strategies may be helpful, this study also suggests the need to introduce or widen existing social welfare programmes towards practically assisting men who may find themselves in economic and financial challenges. This study, though focused on nine men, and may thus not represent the experiences of all the men in Ghana who may undergo similar experiences of economic challenges, insights shed can prompt action that can help prevent suicides among males in such circumstances.

## Reflexivity

The author is a Community Psychologist who values studying human behaviours within contexts. His background may be seen in the foregrounding of the structural challenges and how they shaped relational dynamics and the suicides. This perspective, however, does not in any way reduces the deceased men, and the bereaved persons to cultural sponges who just imbibed what the culture and the social structure imposed on them. Rather, the author ascribes agency to the deceased men and the participants of the study and viewed both as active participants who shaped and were shaped by the social context within which they lived. Again the Author being a Ghanaian just like the deceased men and the bereaved, his interpretation could be bound up in taken-for-granted assumptions on men, masculinity, and suicide. Notwithstanding his background, relying on some colleague experts in qualitative science (who were both Ghanaians and non-Ghanaians) to put critical questions to the analysis at various stages helped the analytic process considerably.

## Conclusion

Economic control and power remain the bedrock of the patriarchal systems in Africa. In Ghana, socially prescribed norms for masculinity include provider roles, being in control, and remaining independent. The current study has highlighted the transient and slippery nature of masculinity in the Ghanaian context. The author argues that the transactional social arrangement in Ghana heighten some men’s distress and elevates suicidal risks when such men lose economic control. Failure to regain control contributes to the state of depending on others, which in itself appeared to be reinterpreted negatively thus heightening distress. The economic challenges produce paradoxes of interdependence and dependence in that the interdependent social ethic enjoins persons in crises to disclose or seek help from close relations, yet for some men, doing so often draws social taunts, which further taints the social image of these men and contributes to suicides. While depending has existential value for men in such crises, and there are some families who are well disposed to providing help for men in economic crises, it appears that for some men who identify strongly with the traditional masculine norm of providing, depending on others emasculates and heightens distress. Thus, the social expectation of men that is connected to men’s social identity constitutes a key risk factor for suicide for men who may be constrained by life adversity. This author strongly recommends public health approaches to male suicide prevention efforts in Ghana and in similar sociocultural contexts. Particularly, exposing the dangers in prevailing masculine norms and promoting broader and yet more constructive norms including emotional expression, help seeking, alongside practical economic intervention for men suffering economic difficulties could be protective against suicides in men
